# The Bonebridge Active Bone Conduction Hearing Implant: Safety, Effectiveness and Outcomes Based on 355 Patients

**DOI:** 10.1111/coa.70050

**Published:** 2025-10-21

**Authors:** Piotr Henryk Skarzynski, Katarzyna Beata Cywka, Emilia Anna Czaplicka, Henryk Skarzynski

**Affiliations:** ^1^ Teleaudiology and Screening Department World Hearing Center, Institute of Physiology and Pathology of Hearing Warsaw Poland; ^2^ Institute of Sensory Organs Kajetany Poland; ^3^ Otorhinolaryngosurgery Clinic World Hearing Center, Institute of Physiology and Pathology of Hearing Warsaw Poland

**Keywords:** bone conduction implants, Bonebridge implantation, conductive hearing loss, hearing loss, mixed hearing loss, single‐sided deafness

## Abstract

**Objectives:**

This study evaluates the safety and efficacy of the Bonebridge BCI 601 and 602 bone conduction implants in our largest cohort to date of 355 patients. The patients had a wide age range and exhibited conductive, mixed, or single‐sided deafness (SSD).

**Design:**

All patients underwent Bonebridge implantation. Pre‐ and post‐implantation evaluations included pure‐tone audiometry, speech recognition tests, and free‐field audiometry. Word recognition was measured using the Polish Monosyllabic Word Test, while speech reception in noise was assessed using the Polish Sentence Matrix Test. Subjective benefit was assessed using the APHAB questionnaire. Follow‐up tests were performed 3–6 months after activation.

**Results:**

Revision surgery was required in 17 patients (4.8%) due to complications, including implant removal in 5 cases. Reimplantation was successful in 4 of these. The APHAB questionnaire showed improved hearing function and all hearing tests also showed significant improvement.

**Conclusion:**

Active bone conduction implantation is an effective method for the rehabilitation of conductive hearing loss, mixed hearing loss, and unilateral deafness. This large cohort study confirms significant hearing improvement and subjective benefits. The low complication rate supports the reliability of the Bonebridge system.

**Summary**

- Rapid technological advances have seen the development of bone conduction implants for patients who do not benefit from or cannot use traditional hearing aids.
- This study, involving 355 patients across a wide age range and various types of hearing loss, marks a significant step forward.
- All patients received Bonebridge implants and were evaluated using objective hearing tests (pure‐tone audiometry, speech recognition, free‐field audiometry) and subjective measures (APHAB questionnaire), with follow‐up assessments conducted 3–6 months post‐activation.
- The audiological results and questionnaire responses confirm the efficacy and safety of the Bonebridge implant in both children and adults.
- The results remained stable over time and the surgical procedure was demonstrably safe.

## 1 Introduction

Hearing is a sense that provides the foundation for verbal communication and active participation in the world around us. According to the World Health Organisation (WHO), in 2021 hearing loss affected approximately 5% of the global population, and by 2050 the number of people affected could exceed 700 million [1]. The scale of difficulties faced by people with hearing loss has, over the years, driven professionals to seek increasingly advanced treatments. Rapid technological advances have seen the development of implantable devices for patients who do not benefit from or cannot use traditional hearing aids [2]. For conductive hearing loss, mixed hearing loss, or single‐sided deafness (SSD), bone conduction devices provide an effective solution [3, 4]. This technology allows mechanical vibrations to be transmitted directly to the inner ear structures, bypassing impairments within the outer or middle ear [3].

The first bone implants were a breakthrough, but they had significant limitations, especially the need to penetrate the skin [5]. The problem has been overcome by the use of fully implantable devices, where the external processor is magnetically attached to the skin, eliminating penetration of tissue [6, 7]. In 2011, Med‐El launched the innovative Bonebridge implant (BCI 601) [6, 8, 9, 10]. This partially implantable hearing aid is designed to treat conductive hearing loss, mixed hearing loss, and unilateral deafness as part of a contralateral routing of signals (CROS) system [6, 11]. A newer model of the implant (BCI 602) was introduced in late 2019. While similar to its forerunner, design modifications enable its use in patients with atypical anatomy.

Although previous studies on Bonebridge implants have demonstrated high efficacy and safety, the small sample sizes involved limit the ability to generalise results to a larger population. In the scientific literature, all analyses have been based on samples of less than 60 patients [12, 13, 14]. This study, involving 355 patients across a wide age range and various types of hearing loss, marks a significant step forward. The large sample size enables a more comprehensive and accurate analysis of the Bonebridge implant's safety and benefits.

## 2 Material and Methods

### 2.1 Study Design

The study used the patients' pre‐operative results as a baseline, and in this sense it was conducted using a prospective design with simultaneous follow‐up. The study protocol was approved by the Bioethics Committee of the Institute of Physiology and Pathology of Hearing (IFPS/KB/7/2020) and adhered to the principles of the Declaration of Helsinki.

The cohort included patients who met the following criteria: age over 6 years, stable bone conduction thresholds within the device manufacturer's recommendations (PTA ≤ 45 dB HL for 0.5–4 kHz), a CT scan confirming adequate anatomical conditions, and awareness by the patient or caregiver of the benefits and limitations of the Bonebridge device.

The implantation site was determined by comparing audiological findings and anatomical conditions in both ears. Before the procedure, all patients (or their parents/caregivers) provided informed consent to participate in the study.

### 2.2 Audiological Assessment

The audiological evaluation included tests before surgery, at activation, and 1 month post‐op, using an Interacoustics AC40 audiometer in a soundproof booth. Pure‐tone audiometry assessed hearing thresholds (125–8000 Hz for air conduction, 250–4000 Hz for bone conduction). Speech intelligibility was measured at 65 dB SPL using the Polish monosyllabic test Damenko and Pruszewicz [15]. A free‐field hearing threshold test with a bone conduction device (Oticon Ponto 5) was conducted, covering 500–4000 Hz, with tones from a speaker 1 m away. Speech audiometry was also done at 65 dB SPL in quiet. To assess speech in noise, the Polish Sentence Matrix Test (PTZM) was used, determining Speech Reception Threshold (SRT) and SNR. An SRT below −2.9 dB SNR indicates normal hearing [16, 17].

Free‐field tests were repeated with the Bonebridge system activated, at both activation and 3–6 months follow‐up, to assess the implant's effectiveness and stability in speech recognition and hearing thresholds.

### 2.3 Questionnaire Assessment

Before the surgery and during the follow‐up visit 3–6 months later, patients completed the Polish version of the Abbreviated Profile of Hearing Aid Benefit (APHAB) questionnaire. This tool assesses the subjective level of difficulty experienced in various listening situations [18].

### 2.4 Surgical Procedure

Before surgery, all patients underwent a CT scan of the temporal bone to assess anatomical conditions. All procedures were performed under general anaesthesia following the standard guidelines provided by the Bonebridge manufacturer. Any contact of the implant with these structures was noted and included in the analysis of results. In cases requiring a reduction in drilling depth, BCI lifts supplied by the manufacturer were used.

Postoperatively, patients remained under observation in the hospital for 3 days. Sutures and dressings were typically removed 7–10 days after implantation. The need for revision and the reasons for revision were analyzed and reported.

### 2.5 Statistical Analysis

Descriptive statistics (mean, standard deviation, minimum, and maximum) were calculated for all variables. The Shapiro–Wilk test was used to assess normality. Chi‐square tests were applied to categorical variables (revision rate, dura contact, and sigmoid sinus contact) to compare device generations (BCI 601 vs. BCI 602). The Mann–Whitney *U* test was performed to compare the contact surfaces of the dura between BCI 601 and BCI 602. Wilcoxon tests were used to compare audiological results with APHAB scores, and a repeated measures ANOVA was used to evaluate differences across time points (preoperative, post‐activation, and follow‐up), with Bonferroni correction applied to all multiple comparisons. Statistical significance was set at *p* < 0.001. Post hoc within‐subject contrast tests identified significant changes over time and assessed the impact of Bonebridge implantation. Analyses were conducted using IBM SPSS Statistics 29.

## 3 Results

### 3.1 Study Group

The study included 355 patients (46.8% male, *n* = 166; 53.2% female, *n* = 189) aged between 5 and 74 years (M = 31.3; SD = 16.7), who underwent implantation of the Bonebridge system (model 601 or 602) between 2020 and 2024. The group included adults and children with different types and causes of hearing loss. The detailed distribution of the results is shown in Table 1.

**TABLE 1:** Patient socio‐demographic and clinical data.

		*n* (%)
Gender	Male	166 (46.8)
	Female	189 (53.2)
Age at the day of Bonebridge implantation	Range	5–74
	M	31.3
	SD	16.7
Implantation year	2020	69 (19.4)
	2021	74 (20.8)
	2022	49 (13.8)
	2023	50 (14.1)
	2024	113 (21.8)
Type of hearing loss	Conductive	178 (50.1)
	Mixed	129 (36.3)
	SSD	48 (13.5)
Side of implantation	Right	193 (54.4)
	Left	162 (45.6)
Implant model	601	69 (19.4)
	602	286 (80.6)
Cause of hearing loss	Microtia with atresia	82 (23.1)
	Atresia	14 (3.9)
	Chronic otitis media	80 (22.5)
	Cholesteatomatous chronic otitis media	69 (19.4)
	Congenital hearing loss	34 (9.6)
	Congenital malformation of the outer and middle ear	29 (8.2)
	Congenital anomaly of the middle ear	14 (3.9)
	Congenital anomaly of the middle and inner ear	7 (2.0)
	Post‐mumps deafness	7 (2.0)
	Acquired overgrowth of the external auditory canal	5 (1.4)
	Congenital anomaly of the inner ear	3 (0.8)
	Progressive deafness, cause unknown	3 (0.8)
	Traumatic skull injury	2 (0.6)

	Otosclerosis	2 (0.6)
	Large vestibular aqueduct syndrome (LVAS)	2 (0.6)
	Acute middle ear infection	1 (0.3)
Middle ear tumour	1 (0.3)

### 3.2 Surgical Intervention

Implantation was performed according to each patient's anatomy and ensuring minimal invasiveness of surrounding structures. The implant was in contact with the dura mater in 52.4% of patients (*n* = 186), covering on average about 40% of the implant area. Contact with the sigmoid sinus was observed in 20.3% of cases (*n* = 72), with exposure in 10.4% (*n* = 37) and compression in 9.9% (*n* = 35).

Revision surgery was required in 17 patients (4.8%). The most common causes were inflammatory lesions with abscess formation and hematoma. In 5 cases, complete implant removal was necessary. The implant was successfully reimplanted in 4 of the 5 patients.

No significant differences were found when comparing BCI 601 and BCI 602 in terms of dura contact (53.6% vs. 52.1%, *χ* ^2^(1) = 0.01, *p* = 0.93), sigmoid sinus contact (21.7% vs. 19.9%, *χ* ^2^(1) = 0.03, *p* = 0.87), or revision rate (7.3% vs. 4.2%, *χ* ^2^(1) = 0.56, *p* = 0.45). Similarly, there was no significant difference in the mean dura contact surface area between the two devices (0.39 vs. 0.43, *p* = 0.69) (Table 2).

**TABLE 2:** Comparison of the surgical outcomes of BCI 601 and BCI 602.

Variable	BCI 601 (*n* = 69)	BCI 602 (*n* = 286)	Test	*p*
Revisions, *n* (%)	5 (7.25%)	12 (4.2%)	*χ* ^2^(1) = 56	0.45
Dura contact, *n* (%)	37 (53.62%)	149 (52,1%)	*χ* ^2^(1) = 0.01	0.93
Sigmoid sinus contact, *n* (%)	15 (21.74%)	57 (19.93%)	*χ* ^2^(1) = 0.03	0.87
Mean dura contact surface, M (SD)	0.39 (0.42)	0.43 (0.46)	*U* (69, 286) = 9583.5; *r* = −0.03	0.69

Abbreviations: *U*, Mann–Whitney *U* test; *χ* ^2^, Chi‐square test.

### 3.3 Pure‐Tone Audiometry

**FIGURE 1:**
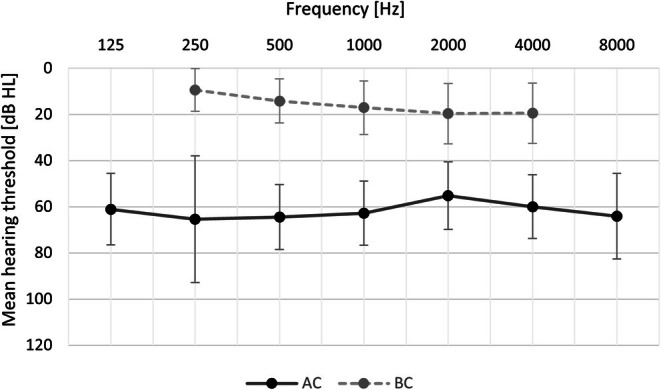
Mean air conduction (AC) and bone conduction (BC) thresholds before treatment. Bars represent standard deviations.

The mean preoperative hearing threshold for air conduction was 61.2 dB HL (SD = 11.6), ranging from 34.3 to 110.6 dB HL; for bone conduction it was 15.7 dB HL (SD = 7.7), ranging from 1 to 46 dB HL. Details are shown in Figure 1.

After surgery, there was no significant change in the mean bone conduction threshold (*Z* = −0.2; *p* = 0.84). Mean hearing thresholds ranged from 1 to 46 dB HL (M = 15.8; SD = 7.7).

### 3.4 Hearing Thresholds With the Bonebridge Implant

**FIGURE 2:**
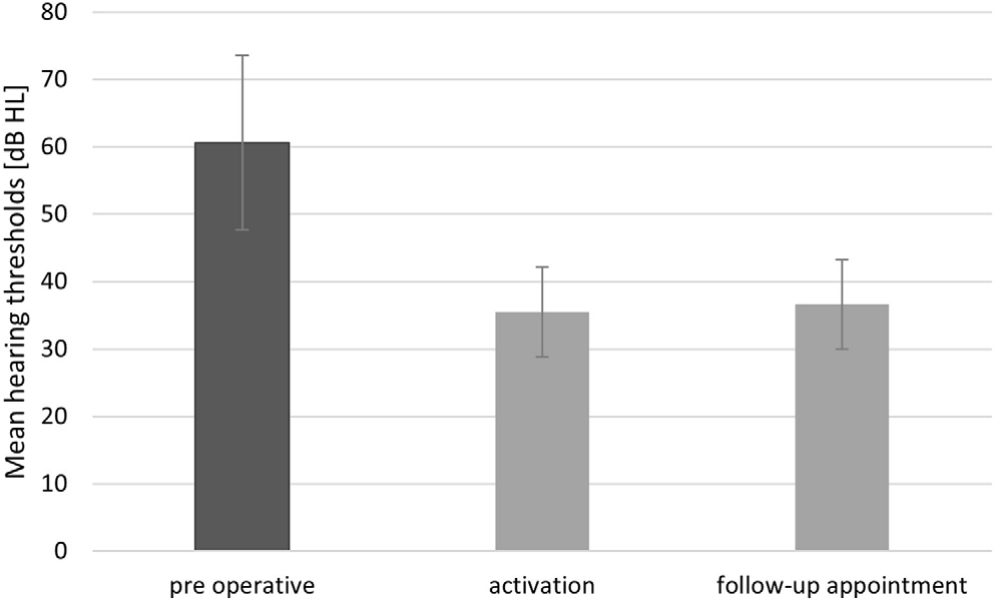
Mean hearing thresholds measured: before implantation, during implant activation, and at the follow‐up visit. The differences between the pre‐implant condition and the two follow‐up measurements are statistically significant (*p* < 0.001). Bars represent standard deviations.

The mean preoperative hearing threshold (500, 1000, 2000, 4000 Hz) was M = 60.6 dB HL (SD = 13). After implant activation, a statistically significant difference in hearing thresholds was observed compared to preoperative results (*F*(2, 281) = 597.63; *p* < 0.001; *e*2 = 0.81). A Bonferroni correction was applied to account for multiple comparisons and all differences remained statistically significant (*p* < 0.001). Mean hearing thresholds at activation were 35.5 dB HL (SD = 6.7) and at follow‐up 36.6 dB HL (SD = 6.6) (Figure 2). Patients with SSD were excluded from the analysis as their hearing thresholds exceeded the audiometer's measurable range.

### 3.5 Speech Discrimination

Before surgery, the mean speech discrimination score measured with headphones was 10.1% (SD = 18.01). The free‐field verbal audiometry score improved significantly to 84.5% (SD = 12.56) between implantation and the time of activation. Further enhancement was observed at follow‐up, with the mean score reaching 92.3% (SD = 7.16).

**FIGURE 3:**
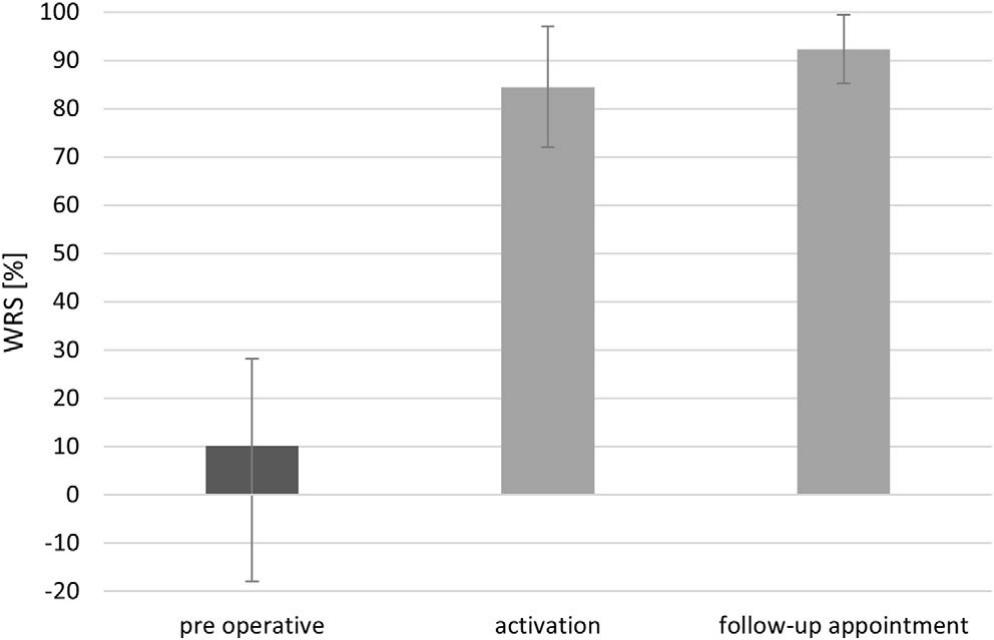
Mean percentage of speech discrimination measured: before implant insertion, during implant activation, and at follow‐up. Differences between measurements are statistically significant (*p* < 0.001). Bars represent standard deviations.

Statistical analysis confirmed a significant improvement in speech understanding after Bonebridge implantation. Repeated measures ANOVA indicated that these differences were highly significant (*F*(2, 287) = 3961.048; *p* < 0.001; *e*2 = 0.932) (Figure 3). A Bonferroni correction was applied for multiple comparisons, confirming that all observed differences remained statistically significant (*p* < 0.001).

### 3.6 Matrix

**FIGURE 4:**
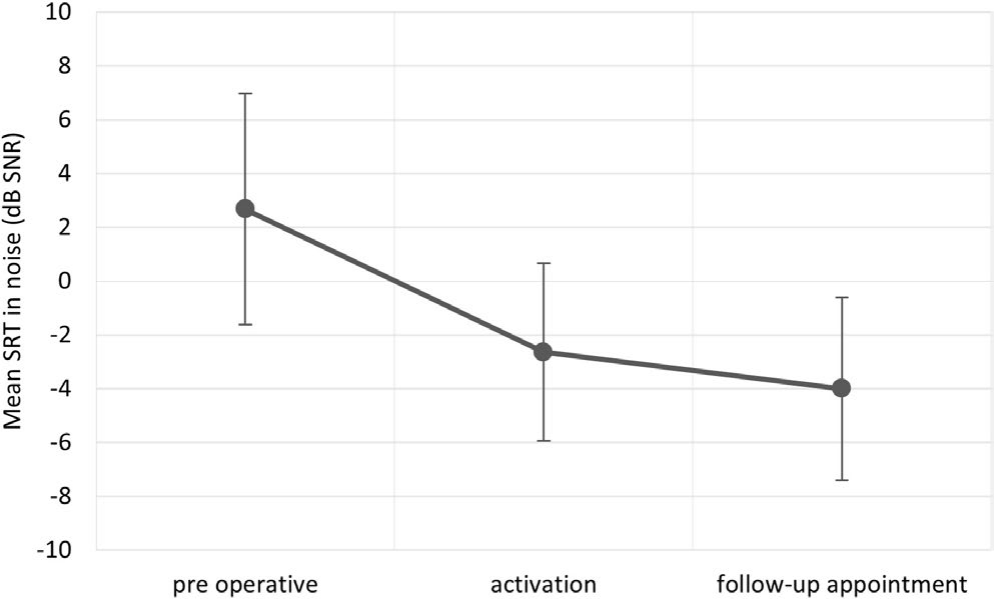
Mean Matrix test score measured: before implant placement, during implant activation, and at the follow‐up visit. Differences between each measurement are statistically significant (*p* < 0.001). Bars represent standard deviations.

The mean Matrix score prior to implant activation was +2.7 dB SNR (SD = 4.34). After activation, the mean score was −2.6 dB SNR (SD = 3.28); at follow‐up, the mean score was −4.0 dB SNR (SD = 3.42) (Figure 4). These differences were statistically significant (*F*(2, 299) = 513.668; *p* < 0.001, *e*2 = 0.631). A Bonferroni correction was applied for multiple comparisons, ensuring that all differences remained statistically significant (*p* < 0.001).

### 3.7 APHAB Questionnaire

**FIGURE 5:**
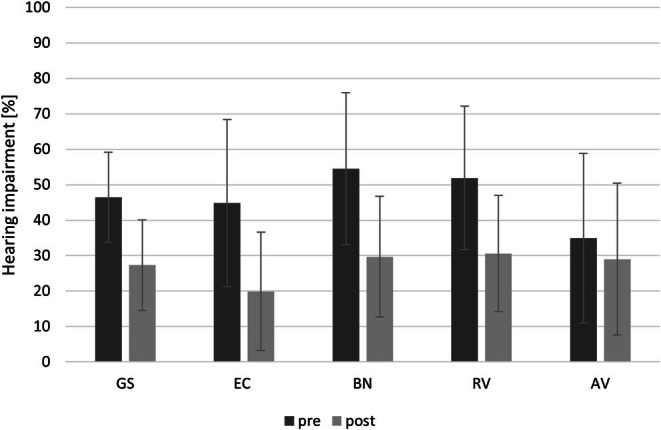
APHAB questionnaire scores. AV: aversiveness; BN: background noise; EC: ease of communication; GS: global score; RV: reverberation. Differences in the scores of each scale are statistically significant (*p* < 0.001). Bars represent standard deviations.

The results of the APHAB questionnaire showed a significant improvement in hearing after Bonebridge implantation. Lower scores on this questionnaire indicate improved hearing quality. Analysis using the Wilcoxon signed rank test showed a statistically significant improvement in the global APHAB score (*Z* = −14.0; *p* < 0.001) over the mean preoperative score (M = 46.3; SD = 17.2), which was significantly higher than the follow‐up score (M = 27.3; SD = 12.8). Significant improvements were also observed in each subscale: EC (*Z* = −13.8; *p* < 0.001); BN (*Z* = −13.7; *p* < 0.001); RV (*Z* = −13.5; *p* < 0.001); and AV (*Z* = −4.4; *p* < 0.001) (Figure 5).

## 4 Discussion

The efficacy and safety of the Bonebridge implants (BCI 601 and BCI 602) have been confirmed multiple times, but never in such a large group of patients. This paper has presented results from 355 patients. The audiological outcomes and patient‐reported measures confirm that both generations of the Bonebridge implant are effective and safe options for children and adults. Although BCI 602 is more widely available, BCI 601 was used in selected cases where anatomical conditions permitted. As BCI 602 can occasionally lead to increased skin tension and related complications, the final decision on which device to use was made during surgery, based on the anatomy of the patient and the surgeon's experience. In our cohort, device selection reflected these factors, with no significant differences observed in revision rates or complication profiles between the two generations.

Sprinzl et al. [12] presented the initial findings on the BCI 602 implant for 33 patients with conductive or mixed hearing loss and SSD. The study revealed notable audiological advancements in thresholds, speech intelligibility, and SRTs, alongside a minimal rate of complications. Post‐operative issues were minor and resolved quickly. Our study produced similar results, with a 4.8% complication rate including five cases requiring reimplantation. The BCI 602 implant improved free‐field thresholds by +25.5 dB, WRS speech intelligibility by +68.0%, and SRTs by −16.5 dB in quiet and by −3.5 dB in noise. Air and bone conduction thresholds remained stable. Dura was exposed in nine patients, with some cases involving sinus exposure or compression [12].

A similar assessment was made in the study by Cywka et al. which analyzed 42 adult patients with conductive or mixed hearing loss who had received the BCI 602 implant [2]. Preoperative results were compared with those obtained during processor activation and at 6 and 12 months after surgery. Air and bone conduction thresholds remained stable over time. The BCI 602 significantly improved free‐field thresholds (+27 dB), WRS speech intelligibility (+74%), and SRT in noise (−6.3 dB SNR).

Another study conducted in the Institute of Physiology and Pathology of Hearing confirmed the safety and benefit of the BCI 602 in children. Among 22 patients aged 8–18, speech understanding improved significantly in both quiet and noise. WRS rose from 12% to 87%, and SRT improved from +4.8 dB to −1.3 dB SNR. APHAB scores also indicated a significant reduction in hearing‐related difficulties [19].

The study by Ratuszniak et al. evaluated the use of the BCI 601 implant in children [20]. The study showed stable thresholds and a functional gain of 30 dB over 12 months, with significant improvements in APHAB scores and speech understanding [20].

The study by Carnevale et al. in 2022 analyzed the outcomes of 52 adult patients with conductive or mixed hearing loss who received a Bonebridge implant [21]. With the Bonebridge implant, the average SRT was 34.2 dB, compared to nearly no correct responses without the implant. At 50 dB, patients with the implant achieved 85% correct responses, while those without achieved just 11% [21].

The effectiveness of the Bonebridge implant has also been assessed in 10 patients with single‐sided deafness (SSD) by Salcher et al. in 2017 [22]. The study included three listening scenarios. Firstly, when there was speech from the front and noise from the side, the speech reception threshold (SRT) improved from −1.8 dB signal‐to‐noise ratio (SNR) unaided to −3.1 dB SNR aided. Secondly, when speech was presented from the implant side and noise from the opposite side, SRT improved from 1.9 dB SNR (unaided) to −0.64 dB SNR (aided). Finally, with speech only from the implant side, SRT improved from 34.5 dB SPL unaided to 32.3 dB SPL aided. The implant significantly improved SNR in all scenarios [22].

In 2022, Ratuszniak et al. [23] presented the results of one of the larger groups of Bonebridge implant patients published to date. The work evaluated the use of the Bonebridge implant in 81 adult patients (25 with CHL, 36 with MHL, 20 with SSD). APHAB scores showed a significant improvement across all groups. Satisfaction ratings were mixed: 60% rated sound quality as fair to poor, 50% had neutral feelings about satisfaction, and 40% felt that the improvement was significant. As in previous studies, SSD patients reported lower satisfaction and less perceived improvement than CHL and MHL patients [23].

Finally, a study by Kim et al. [9] in 2017 on patients with unilateral deafness demonstrated that it is important to ensure patients understand that, while bone conduction implants improve speech understanding in noisy environments, they do not fully restore sound source localization [9].

The findings of this study align with those of previous studies. Bonebridge implants have been shown to provide clear, lasting hearing benefits. All reports show improved hearing and speech comprehension in various acoustic settings, alongside high patient satisfaction and fewer everyday hearing difficulties. Although the average free‐field threshold was 36.6 dB HL—below normal—patients still achieved excellent speech comprehension in quiet and noisy environments. Sound processor settings were tailored using tonal audiometry, vibrogram results, and patient feedback.

## 5 Conclusions

Active bone conduction implantation is an effective method for the rehabilitation of conductive hearing loss, mixed hearing loss, and SSD. Following implantation, all our patients experienced improved hearing and speech understanding in both quiet and noisy environments. Subjectively, the device alleviated the daily challenges associated with hearing loss and enabled effective verbal communication even in difficult acoustic conditions. The results remained stable over time, and the surgical procedure was demonstrably safe.

## Author Contributions

P.H.S. made substantial contributions to the conception and design of the work and to the acquisition of data, revised it and made final approval of the version to be published. K.B.C. made substantial contributions to the conception and design of the work, drafted the manuscript and contributed to data interpretation. E.A.C. contributed to data analysis and interpretation. H.S. revised the work and made final approval of the version to be published. All authors (P.H.S., K.B.C., E.A.C., and H.S.) approved the final version to be published and agree to be accountable for all aspects of the work in ensuring that questions related to the accuracy or integrity of any part of the work are appropriately investigated and resolved.

## Conflicts of Interest

The authors declare no conflicts of interest.

## Data Availability

The data that support the findings of this study are available from the corresponding author upon reasonable request.
